# Mulberry polyphenol extracts attenuated senescence through inhibition of Ras/ERK via promoting Ras degradation in VSMC

**DOI:** 10.7150/ijms.64763

**Published:** 2022-01-01

**Authors:** Ching-Pei Chen, Kuei-Chuan Chan, Hsieh-Hsun Ho, Hui-Pei Huang, Li-Sung Hsu, Chau-Jong Wang

**Affiliations:** 1 Cardiovascular division of Changhua Cristian Hospital, Changhua, Taiwan.; 2 School of Medicine, Chung Shan Medical University, Taichung 402, Taiwan.; 3 Department of Internal Medicine, Chung Shan Medical University Hospital, Taichung, 402, Taiwan.; 4 Institute of Biochemistry and Biotechnology, Chung Shan Medical University, Tai-chung, 402, Taiwan.; 5 Department of Biochemistry, School of Medicine, Medical College, Chung Shan Medical University, Taichung, 402, Taiwan.; 6 Institute of Medicine, Chung Shan Medical University, Taichung, 402, Taiwan.; 7 Clinical Laboratory, Chung Shan Medical University Hospital, Taichung, 402, Taiwan.; 8 Department of Health Diet and Industry Management, Chung Shan Medical University, Taichung 402, Taiwan.; 9 Department of Medical Research, Chung Shan Medical University Hospital, Taichung, 402, Taiwan.

**Keywords:** mulberry polyphenol extract, Ras, smooth muscle cell, senescence

## Abstract

Ageing is one of the major risk factors of human diseases, including cancer, diabetes, and cardiovascular disease. Mulberry exhibits a wide range of functions, such as anti-oxidant, anti-inflammation, and anti-diabetes. In this study, we investigated the role of mulberry polyphenol extract (MPE) in K-Ras-induced senescence of smooth muscle cells. Forced expression of K-Ras enhanced senescence of smooth muscle A7r5 cells as shown by the elevation of β-galactosidase activity. Treatment with MPE significantly repressed the Ras, phosphorylated ERK, and β-galactosidase level. MPE triggered the association of cyclins with their corresponding cyclin-dependent protein kinases and hyperphosphorylated retinoblastoma (RB). MPE also down-regulated the levels of K-Ras-induced CDK inhibitors. MPE enhanced the phosphorylated AMP-dependent protein kinase (AMPK) and inducible nitric oxide synthase (iNOS) level in the presence of K-Ras. Pretreatment with either L-NAME or AMPK inhibitor reversed the effects of MPE. In addition, L-NAME and AMPK inhibitor repressed the MPE-induced total and phosphorylated 3-hydroxy-3-methylglutaryl coenzyme A (HMG-Co A) level. MPE repressed K-Ras-induced G0/G1 arrest, whereas L-NAME and AMPK inhibitor blocked the effects of MPE. Our results indicated that MPE recovered the K-Ras-induced senescence of vascular smooth muscle cells through iNOS and AMPK-dependent pathway. Our findings suggested that MPE may prevent ageing-induced atherosclerosis.

## Introduction

Ageing is a multiple factor-induced process. Lopez-Otin et al. have categorized nine hallmarks of ageing, namely, genomic instability, telomere attrition, epigenetic alterations, loss of proteostasis, deregulated nutrient sensing, mitochondrial dysfunction, cellular senescence, stem cell exhaustion, and altered intercellular communication 1. Ageing promotes a wide range of human diseases, such as atherosclerosis, neurodegeneration diseases, and cancers 1. Scientists focus on developing novel agents to attenuate the ageing process and prevent ageing-related diseases.

Ras proteins play a critical role in several physiological functions, such as cell proliferation, migration, and ageing process 2. Sun et al.^3^ demonstrated that mutation of RAS1 or RAS2 promoted the life span in *Saccharomyces cerevisiae.* In fruit fly *Drosophila melanogaster*, a constitutively activated form of Anterior open (AOP), a transcriptional factor repressed by RAS, contributed to a longer lifespan 4. Jazwinski et al. demonstrated that genetic variants of HRAS1, apolipoprotein E, and ceramide synthase LASS1 synergistically play a critical role in the longevity and healthy ageing of humans 5.

Recent emerging reports indicated that extracts from traditional herbs slowed the ageing process and treated ageing-related diseases. Exposure to ethanol extracts from the leaves of *Humulus japonicus* increased the lifespan of yeast cells through the enhanced expression of longevity-related proteins, such as sirtuin 1 and AMP-dependent protein kinase 6. Administration of ethyl acetate extracts from *Physalis alkekengin* (PAE) significantly reversed D-galactose-induced ageing-related impairment of neuron functions 7. Moreover, PAE also attenuated D-galactose triggered senescence by diminished oxidative stress 7. Treatment with coffee silverskin aqueous extract clearly inhibited tert-butyl hydroperoxide-induced ageing in HaCaT cells and promoted longer longevity in *Caenorhabditis elegan* 8*.* Mulberry (*Morus alba* L.) is an economically important plant that is widely distributed in Asia region and extracts from different parts of mulberry enriched in flavonoids possess several biological functions, such as anti-oxidant, anti-obesity, and anti-inflammation 9. Mulberry leaf extract (MLE) prevented atherosclerosis through the reduction of oxidative low density lipoprotein-elevated reactive oxygen species 10. Administration of MLE attenuated the obesity-induced non-alcohol fatty liver disease through the regulation of lipogenesis and the elevation of anti-oxidant enzyme expression 11. A previous report from our laboratory indicated that mulberry water extract induced smooth muscle cells to undergo apoptosis via the activation of both intrinsic and extrinsic pathways and inhibited atherosclerosis in a rabbit model 12. Moreover, MPE mitigated the migration of A7r5 cells through the downregulation of FAK/SRC/PI3-K pathway 13. The molecular mechanisms of MPE on Ras-induced VSMC ageing still remains to be elucidated. In the present study, we investigated the effect of MPE on Ras-induced ageing in A7R5 smooth muscle cells.

## Materials and Methods

### Materials

All chemicals including AMPK inhibitor (Compound C) and L-NAME were purchased from Sigma-Aldrich (St. Louis, MO, USA). Antibodies against p53 (Pab 240), retinoblastoma (RB; G3-245) and E2F (KH95/E2F) were purchased from BD Biosciences (San Jose, CA, USA). Phosphorylate p53 (16G8) and phosphorylated RB (D20B12) antibodies were obtained from Cell Signaling Technology Inc. (Beverly, MA, USA). Anti-phosphorylated ERK (E-4), anti-ERK (C-14), anti-cyclin D1 (HD-11), anti-cyclin E (M-20), anti-cyclin A (H-432), anti- CDK2 (D-12), anti- CDK4 (DSC-35), anti- p21 (F-5), anti- p27 (F-8), and anti- p16 (F-12) were purchased from Santa Cruz Biotechnology Inc. (Santa Cruz, CA, USA).

### Preparation of MPE and HPLC analysis

MPE was prepared as previously reported 13. Dried mulberry fruit powder (100 g) was dissolved in 500 ml methanol and stirred at 50 °C for 3 h. The debris of methanol extract was removed by filter. The extract was lyophilized under reduced pressure at room temperature. After resuspension in 500 mL of 50 °C distilled water and extraction with 180 mL of ethyl acetate thrice, the MPE was redissolved in 300 mL of distilled water and lyophilized. The MPE powders were stored in -80 °C until use. HPLC analysis of MPE was performed as previously reported 13. The major compounds of MPE included rutin (18.17%), protocatechuic acid (13.76%), naringenin (6.71%), epigallocatechin gallate (6.26%), caffeic acid (6.15%), quercetin (5.97%), and several minor polyphenols, such as epicatechin (4.69%), catechin (3.19%), gallic acid (2.67%), p-coumaric acid (2.48%), and hesperetin (2.08%) 13.

### Cell culture and selection of stable clones

The rat smooth muscle A7r5 cells obtained from American Type Culture Collection (ATCC, Manassas, VA, USA) were maintained in Dulbecco's modified Eagle's medium (DMEM; HyClone, Marlborough, Massachusetts, USA) supplemented with 10% fatal bovine serum (Biological Industries, Kibbutz Beit-Haemek, Israel), 1% glutamine (HyClone, Marlborough, MA, USA), and 1% penicillin-streptomycin (HyClone, Marlborough, MA, USA). The cells were cultured in an incubator at 37 °C with a humidified atmosphere of 5% CO_2_.

The A7r5 cells were seeded in 6-well plate and transfected with pcDNA3.0 or pcDNA3.0-K-Ras plasmid by Lipofectamine 2000 (ThermoFisher SCIENTIFIC, Inc. Waltham, MA, USA) according to the manufacturer's recommendation. A7r5 cells with a stable expression of K-Ras were selected by G418 (ThermoFisher SCIENTIFIC, Inc. Waltham, MA, USA).

### β-galactosidase activity assay

The A7r5 cells expressing or not expressing K-Ras were seeded in 6-well plate at a density of 3 × 10^5^. At days 2, 4, 8, 12, and 16, the cells were fixed by a fix solution (2% formaldehyde and 0.2% glutaraldehyde) at room temperature for 5 min. After washing with phosphate buffered saline (PBS), cells were incubated with the staining solution (40 mM citric acid, sodium phosphate pH 6.0, 150 mM NaCl, 2 mM MgCl2, and 20 mg/ml X-gal) at 37 °C for 12-16 h. Images of the cells were taken using microscopy at X 400 magnification.

### Cell cycle analysis

The A7r5 cells expressing or not expressing K-Ras were treated with MPE in the presence of L-NAME or AMPK inhibitor for 24 h. Cells were detached and fixed by 70% alcohol overnight at -20 °C. After washing with cold PBS, cells were incubated with 50 μg/mL propidium iodine (PI) and 100 μg/ml RNase A in PBS for 15 min in the dark. The cell cycles of stained cells were analyzed by flow cytometer (Becton Dickinson, CA, USA) and CellQuest Software (Becton Dickinson, CA, USA).

### Immunoprecipitation assay

The A7r5 cells were lysed by RIPA buffer containing proteinase inhibitors (1 mM/ml Na3VO4, 1.7 μg/ml leupeptin and 100 μg/ml PMSF). The protein concentration was measured by Bio-Rad protein assay kit. Cell lysate (500 mg) was incubated with indicated antibodies (1:100 dilution) plus 30 μl Agarose-G at 4 °C overnight. The complexes were washed with PBS twice and centrifuged at 8000 rpm for 5 min. The immunocomplexes were subjected to Western blot analysis.

### Western blot analysis

Proteins (50 μg) were separated by sodium dodecyl sulfate polyacrylamide gel electrophoresis (SDS-PAGE) and then transferred into polyvinylidene fluoride (PVDF) membrane. The membrane was blocked by phosphate buffered saline (PBS) containing 5% non-fat milk for 1 h and incubated with specific antibodies for overnight at 4 °C. The membrane was washed with PBS containing 0.1% Tween-20 thrice and then incubated with an horseradish peroxidase (HRP)-conjugated second antibody. The positive signals were determined by using an enhanced chemiluminescence kit.

### Reverse transcription-polymerase chain reaction (RT-PCR)

The TRIzol reagent was utilized to purify total RNA from A7r5 cells with or without K-Ras transfection in the presence or absence of MPE for indicated time according to the manufacturer's protocols. First strand cDNA was generated by Moloney Murine Leukemia Virus (M-MLV) reverse transcriptase, oligo-dT primer and 4 μg total RA. PCR was performed using K-Ras primers were: Sense: 5'- CTTGATAATCTTGTGTGGAAC-3' and Antisense: 5' -CCTCCCTTTACAAATTGTAC- 3'. GAPDH primers were: sense: 5'-ACCACAGTCCATGCCATCAC-3 and anti-sense: 5'-TCCACCACCCTGTTGCTGTA-3'The PCR condition was: denaturation at 95 ^o^C for 1 min, annealing at 55^o^C for 1 min, and extension at 72 ^o^C for 1 min. Total cycle was 30 for K-Ras and 28 for GADPH. The PCR products were separated by agarose gel and pictured.

### Statistical analysis

Data were represented as means ± standard division obtained from three independent experiments. Data were analyzed by Student t test using SPSS software version 12 (IBM, Armonk, NY, USA). Significant difference was set as P < 0.05.

## Results

### Overexpression of K-Ras-induced senescence in smooth muscle cells

**Figure 1:**
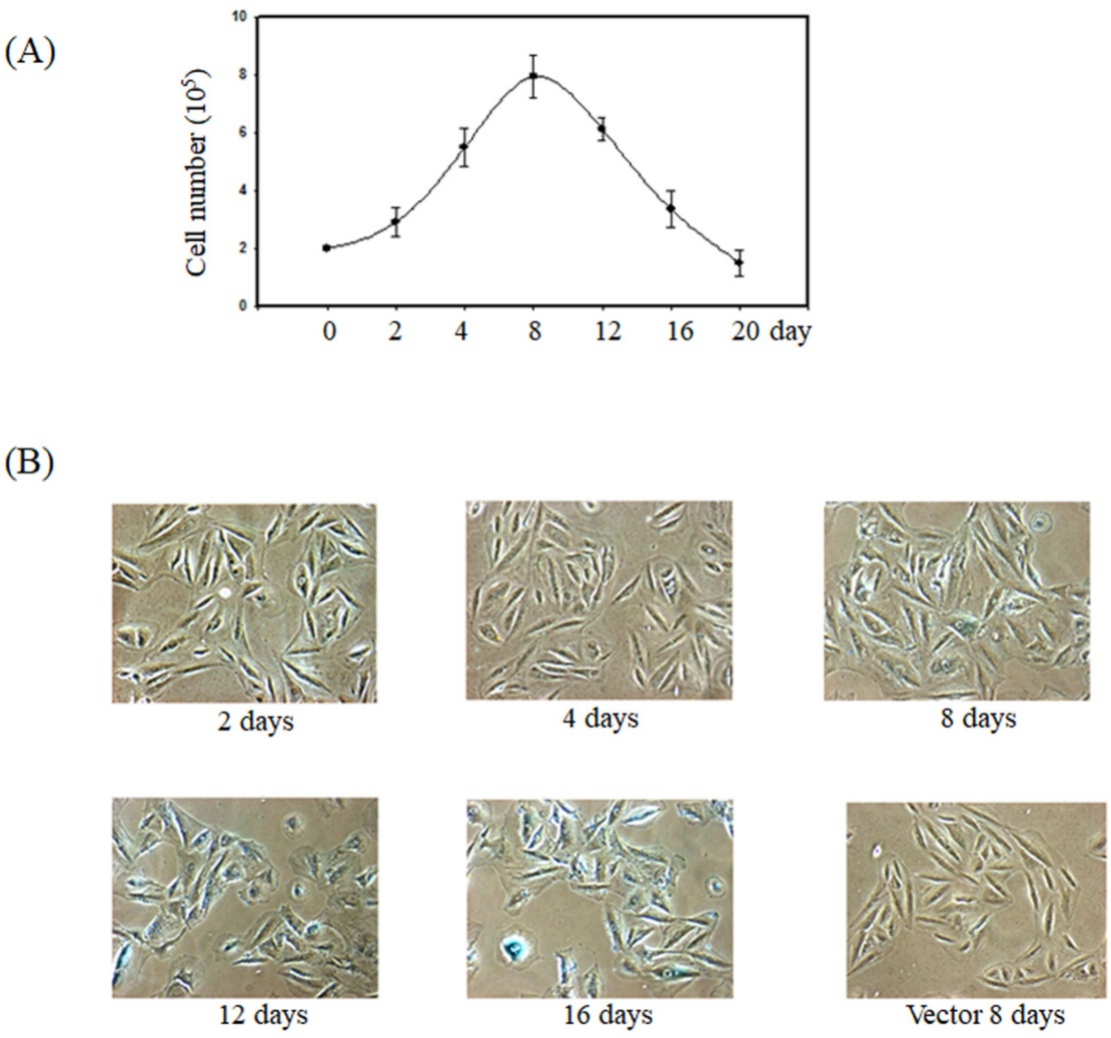
** Effect of K-Ras on cell growth and senescence of A7r5 cells.** A7r5 cells were with transfected K-Ras and selected by G418 medium for 14 days. (A)The cell were seeded in 6 well plate and maintained for indicated time course. Cell numbers were measured by cell counter. (B) The senescence was detected by β-galactosidase assay.

Previous report showed the forced expression of K-Ras triggered senescence in smooth muscle cells via the activation of the p53 and ERK pathways 15. To verify whether K-Ras-induced senescence in smooth muscle cells, we established the smooth muscle A7r5 cell's stable expression of K-Ras. The cell numbers increased before day 8 and significantly decreased after day 12 in the group stably expressing K-Ras compared with the normal control (Fig. 1A). In addition, the ageing marker (β-galactosidase) activity was detected at day 8 and clearly increased at days 12 and 16 compared with the vector alone in A7r5 cells (Fig. 1B).

### MPE attenuated Ras-induced senescence and signals

**Figure 2:**
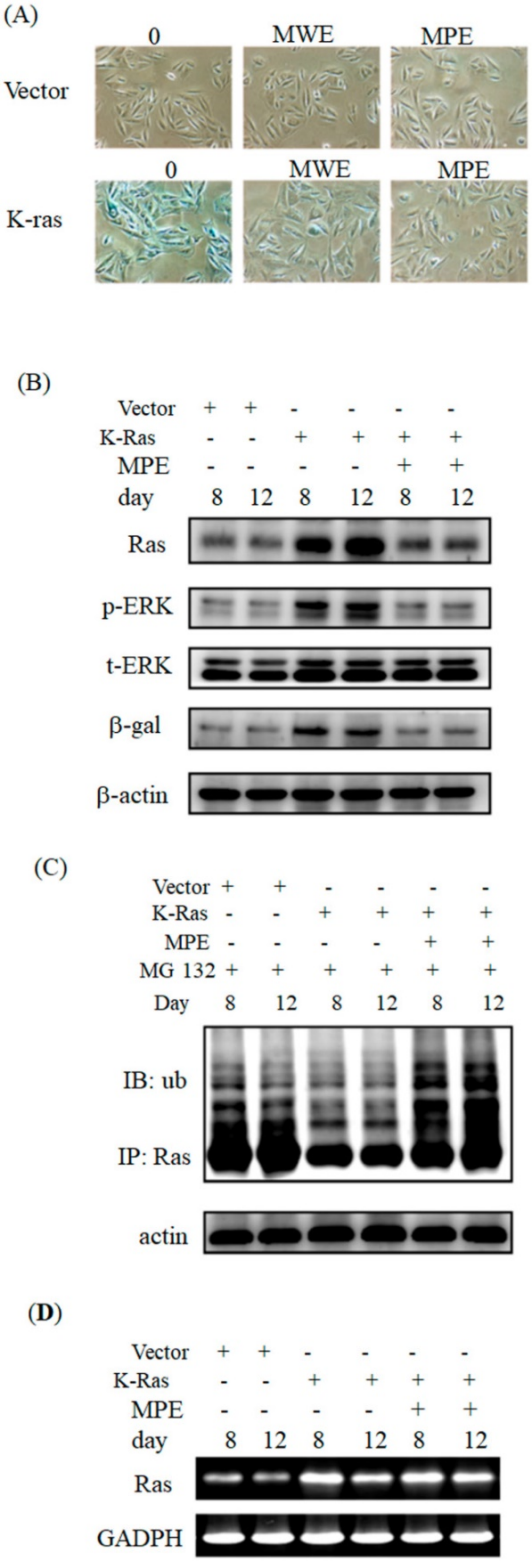
** MPE blocked K-Ras-induced senescence and downstream Ras signals of A7r5 cells.** (A) A7r5 cells were transfected with K-Ras and treated with MWE or MPE at indicated concentration for 12 days and senescence level was detected by β-galactosidase assay. (B) Western blot analysis was conducted to detect the expresso of indicated proteins expression of A7r5 cells and stable expressed K-Ras A7r5 cell with or without MPE treatment at day 8 and 12. (C) A7r5 cells and stable expressed K-Ras A7r5 cell with or without MPE treatment at day 8 and 12 in the presence or absence of MG-132 were subjected to perform immunoprecipitation by anti-Ras antibody and Western blot with anti-Ras antibody. Actin was used as internal control. (D) Total RNAs derived from A7r5 cells and stable expressed K-Ras A7r5 cell with or without MPE treatment at day 8 and 12 were subjected into RT-PCR analysis of K-Ras. GAPDH was used as internal control.

Next, we investigated the effects of MPE on K-Ras-induced ageing in A7r5 cells. Exposure to 0.005 mg/mL of MPE obviously decreased β-galactosidase activity in A7r5 cells stably expressing K-Ras at day 12 (Fig. 2A). The effect of MPE was stronger than MWE. Thus, we selected MPE for further studies.

To address the effects of MPE on the expression of Ras and its signals, we performed Western blot analysis. Ras expression clearly increased at days 8 and 12. In parallel, phosphorylated ERK and β-galactosidase levels increased in K-Ras-expressing cells. MPE significantly reduced Ras, phosphorylated ERK, and β-galactosidase expressions (Fig. 2B).

To address whether MPE enhanced the degradation of Ras, immunoprecipitation and Western blot analysis were performed. As shown in Fig 2C, significant ubiquitination was found in the presence of MG-132 and MPE co-treatment groups.

Moreover, we also conducted RT-PCR to detect the mRNA level of K-Ras in the presence of MPE. The mRNA level was increased in K-Ras overexpressd A7r5 cells whereas MPE slightly reduced the K-Ras mRNA at day 8 post-transfection (Figure 2D).

### MPE reversed K-Ras-induced cell cycle-related proteins' interaction and expression

**Figure 3:**
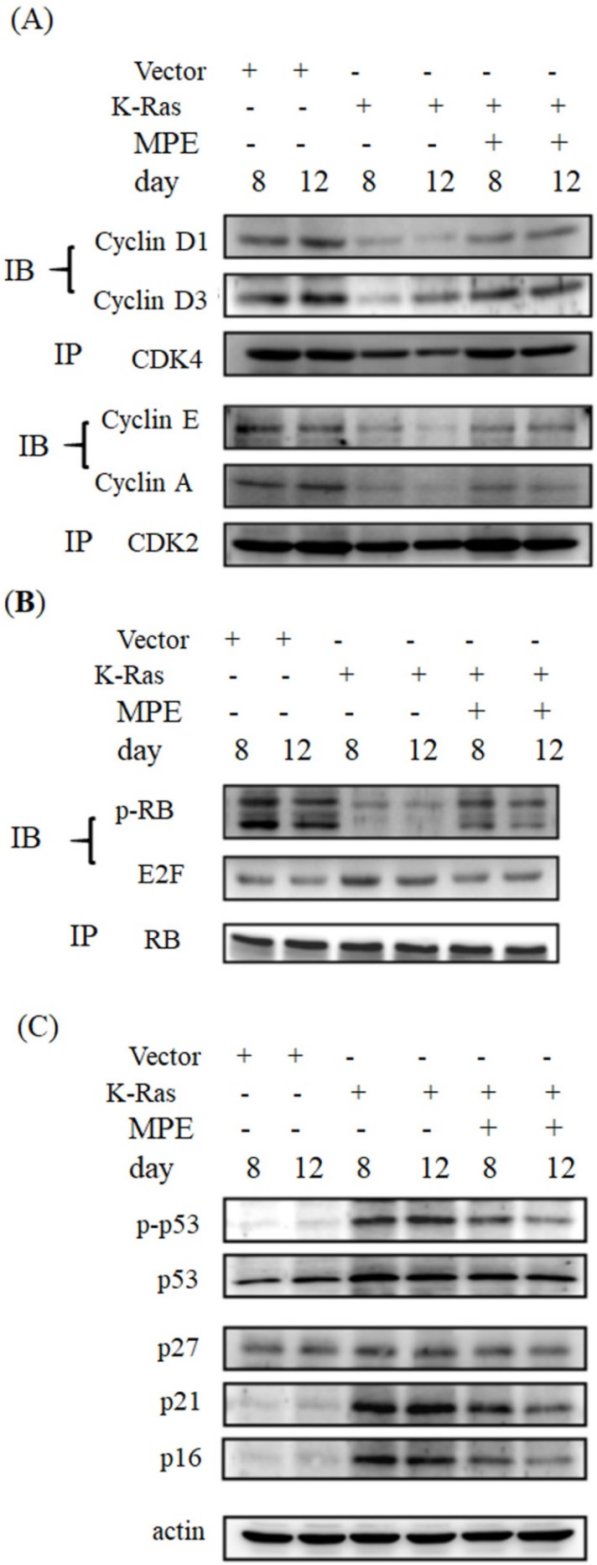
** MPE affected cell cycle-related proteins expression.** (A) Protein extracts derived from A7r5 cells and stable expressed K-Ras A7r5 cell with or without MPE treatment at day 8 and 12 were subjected into immunoprecipitation with anti-CDK4 (upper panel) or anti-CDK2 (low panel) and then performed Western blot analysis using indicated antibodies. (B) Western blot analysis of indicated CDFK inhibitors of protein extracts derived from A7r5 cells and stable expressed K-Ras A7r5 cell with or without MPE treatment at day 8 and 12.

Cell cycle arrest is one of the hallmarks of ageing. To determine whether MPE affected the expressions of cell cycle-related proteins, immunoprecipitation and Western blot were conducted. As shown in Fig. 3, the interaction of cyclin-dependent protein kinase 4 (CDK4) with cyclin D1 or D3 was clearly reduced in K-Ras-expressing cells whereas co-treatment with 0.005 mg/mL MPE enhanced the interaction of CDK4 with cyclin D1 or D3. Similarly, the interaction of CDK2 with cyclin A or E was blocked by K-Ras whereas MPE reversed the interaction. K-Ras diminished phosphorylation of RB and enhanced the association of E2F with RB. Treatment with MPE enhanced the phosphorylation of RB and promoted the dissociation of RB and E2F. Moreover, the increased p53 phosphorylation and enhanced expression of CDK inhibitors, such as p27, p21, and p16, by K-Ras was reversed by MPE (Fig. 3).

### MPE affected AMP-dependent protein kinase (AMPK) and inducible nitric oxide synthase (iNOS) expressions

**Figure 4:**
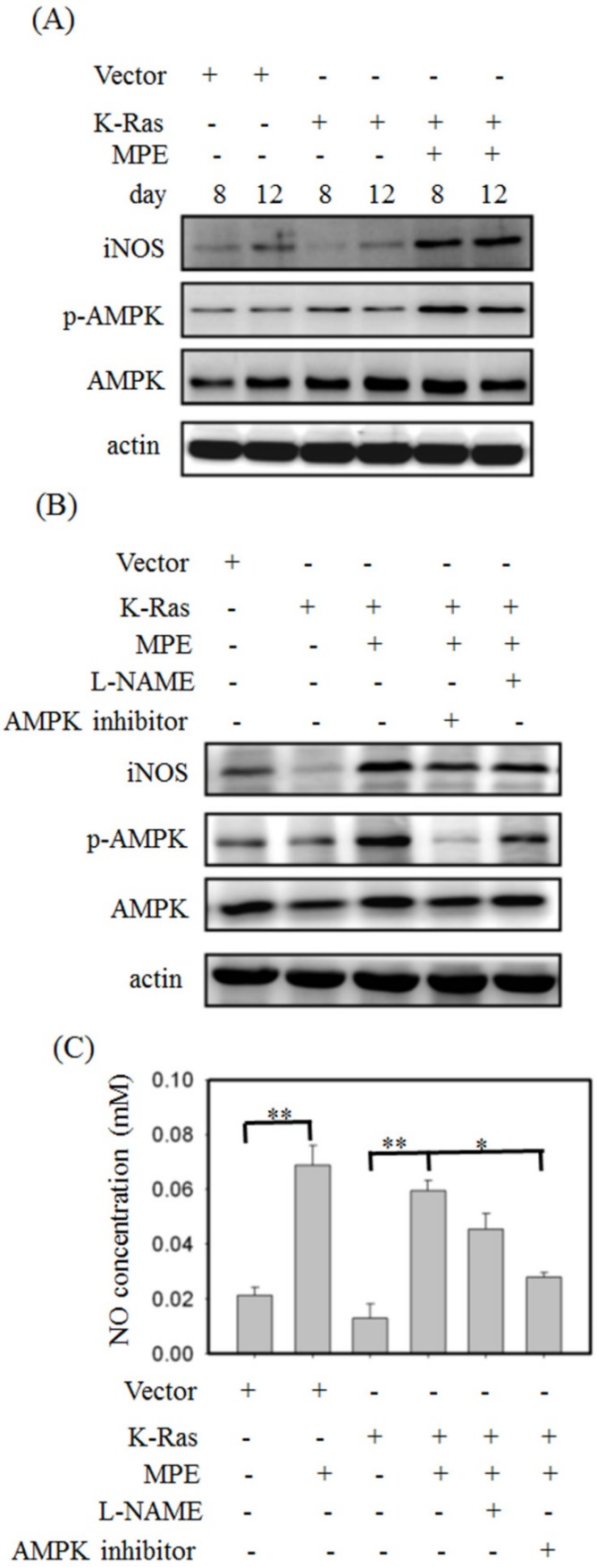
** MPE enhanced the activation of AMPK and iNOS.** (A) Protein extracts derived from A7r5 cells and stable expressed K-Ras A7r5 cell with or without MPE treatment at day 8 and 12 were subjected into Western blot analysis using antibodies against iNOS, phosphorylated AMPK (p-AMPK), and AMKP. (B) Protein extracts derived from A7r5 cells and stable expressed K-Ras A7r5 cell with or without MPE in the presence of L-NAME or AMPK inhibitor were subjected into Western blot analysis using antibodies against iNOS, phosphorylated AMPK (p-AMPK), and AMKP. Anti-actin was used as internal control. (C) The nitric oxide concentration of A7r5 cells and stable expressed K-Ras A7r5 cell with or without MPE in the presence of L-NAME or AMPK inhibitor were detected. Data represented means standard division from at least three independent experiments. 8 denoted P < 0.05; ** denoted P < 0.001.

To detect the effect of MPE on AMPK and iNOS expressions, Western blot analysis was performed. No overt alternation of phosphorylated AMPK and iNOS level was found in K-Ras-expressing A7r5 cells compared with control A7R5 cells. MPE clearly increased the phosphorylated AMPK and iNOS levels in A7r5 cells stably expressing K-Ras (Fig. 4A). Pretreatment with iNOS inhibitor (L-NAME) or AMPK inhibitor reversed the MPE-induced effects (Fig. 4B). Similarly, MPE-induced NO concentration was blocked by the L-NAME and AMPK inhibitor (Fig. 4C).

### AMPK and iNOS inhibitor attenuated the effects of MPE

**Figure 5:**
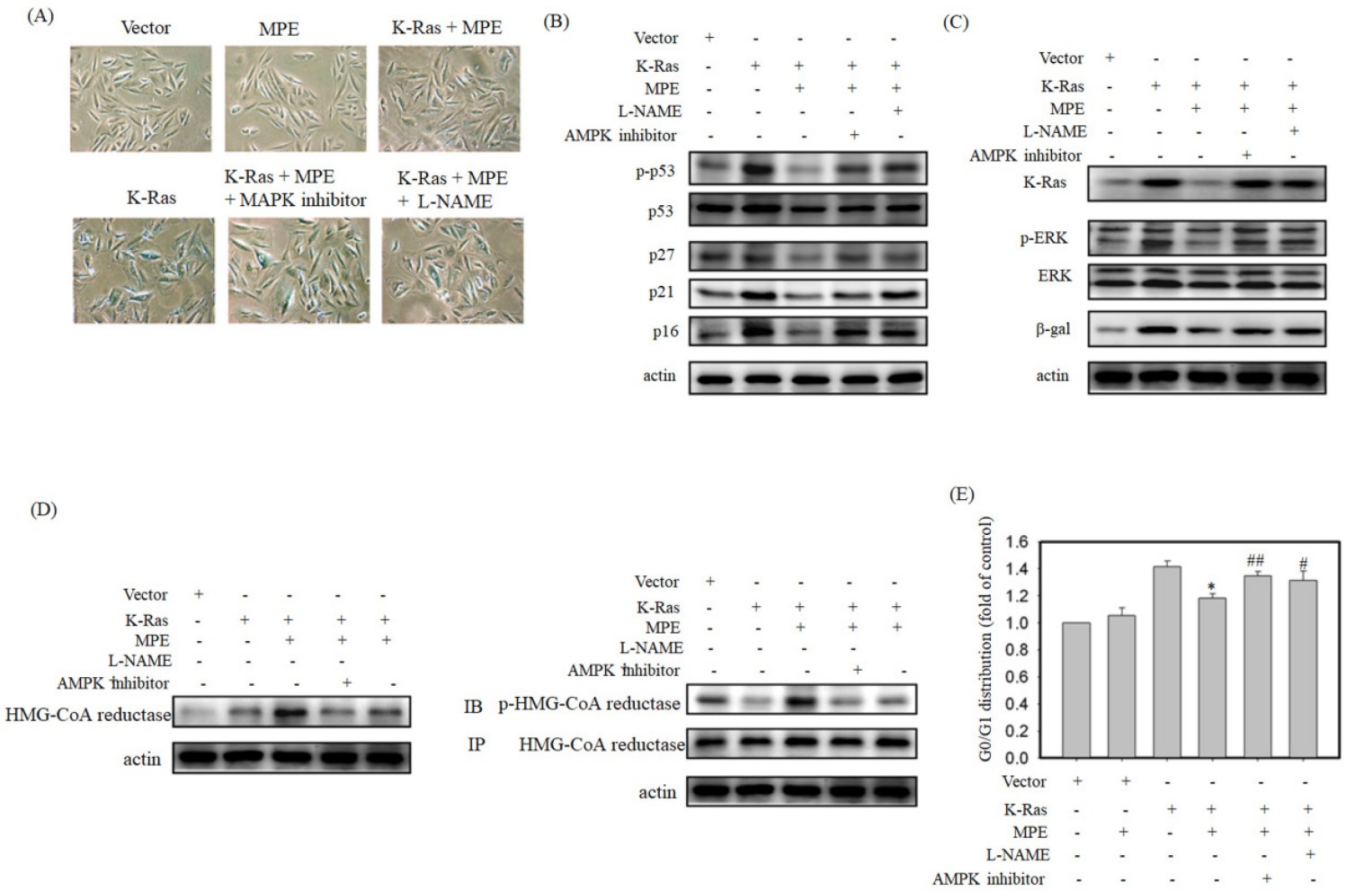
** iNOS inhibitor (L-NAME) or AMPK inhibitor blocked anti-senescence of MPE in K-Ras overexpressed A7r5 cells.** (A) β-galactosidase activity was performed in K-Ras stable expressed A7r5 cells or parental cells with or without MPE treatment and indicated inhibitors. (B and C) Protein extracts derived from A7r5 cells and stable expressed K-Ras A7r5 cell with or without MPE in the presence of L-NAME or AMPK inhibitor were subjected into Western blot analysis using indicated antibodies. Anti-actin was used as internal control. (D) Expression and phosphorylated HMG-Co A reductase was analyzed by Western blot and immunoprecipitation, respectively. (E) The G0/G1 distribution of A7r5 cells and stable expressed K-Ras A7r5 cell with or without MPE in the presence of L-NAME or AMPK inhibitor was detected by flow cytometry analysis. Data represented means standard division from at least independent experiments. * denoted P < 0.05 compared to stable expressed K-Ras A7r5 cell. ## and # denoted P < 0.001 and 0.05 compared to stable expressed K-Ras A7r5 cell with MPE treatment.

To verify the effects of AMPK and NOS inhibitor on K-Ras-induced ageing in the presence of MPE,β-galactosidase activity was determined, and Western blot analysis was performed. Increasedβ-galactosidase activity was found in K-Ras-expressing A7r5 cells treated with AMPK and NOS inhibitor in the presence of MPE (Fig. 5A). MPE's blockage of K-Ras-induced phosphorylated p53, total p53, p27, p21, and p16 expressions were reversed by AMPK inhibitor and L-NAME (Fig. 5B). Moreover, MPE also recovered the K-Ras, phosphorylated ERK, and β-galactosidase expressions (Fig. 5C). In addition, MPE elevated the expression and phosphorylated status of HMG-CoA reductase whereas AMPK and iNOS inhibitors reversed the phenomenon (Fig. 5D).

Cell cycle analysis was used to detect the effects of AMPK and NOS inhibitor on MLE repressed cell cycle arrest. As shown in Fig. 5E, the stable expression of K-Ras in A7r5 cells induced 1.4-fold of G0/G1 arrest compared with the control. Exposure to MPE attenuated the arrest to 1.1-fold, whereas co-treatment with AMPK or NOS inhibitor recovered the cell cycle arrest to 1.3-fold in the presence of K-Ras.

## Discussion

Ageing is one of the major risk factors for atherosclerosis, dementia, and cancer. Prevention of the ageing process is an emerging health issue. Supplementing the diet with flavonoids has been shown to attenuate the ageing process. In this study, we demonstrated that MPE diminished K-Ras-induced ageing in vascular smooth muscle A7r5 cells. MPE significantly attenuated the expression and downstream targets of K-Ras in A7r5 cells. MPE promoted the progression of cell cycle through the enhancement of CDK activities and the downregulation of CDK inhibitors, such as p53, p21, and p16. Moreover, AMPK and iNOS were involved in MPE-mediated functions.

Ras family proteins play pivotal roles in diverse cellular functions, including cell proliferation, cell differentiation, senescence, and metastasis 2. Knockdown of RAS1 and RAS2 prolonged the replication and chronological lifespan in yeast, respectively 3. In human smooth muscle cells, activated Ras ((H-rasV12) significantly induced senescence, as evidenced by the elevated senescence-associated β-galactosidase activity 14. Futami et al. 15 demonstrated that the forced expression of wild-type or constitutively activated hras in zebrafish Epithelioma papulosum cyprinid (EPC) cells caused senescence by increasing the p53 expression and β-galactosidase activity. In line with these observations, our results demonstrated that K-Ras obviously enhanced β-galactosidase activity and triggered senescence in A7r5 cells.

Using traditional Chinese medicine as anti-ageing molecules has received attention. Mulberry leaf polyphenol affected the transcriptional factors, such as DAF-12, DAF-16, PHA-4, and NHR-80; it controlled the downstream targets expression and eventually prevented ageing in C. elegans 16. Administration of black mulberry extracts diminished oxidative stress and alleviated cognitive impairment in D-galactose-induced ageing mice 17. The major components in mulberry extracts also delayed the ageing process. Administration of rutin significantly delayed diabetes-induced ageing in rat CS collagen 18. Rutin suppressed lipid peroxidation and reduced inflammation in D-glucose-induced ageing mice 19. In old fibroblast cells, quercetin significantly increased the expression of anti-ageing genes, including growth differentiation factor 11 (GDF11) and Sirt1 20. In the present study, we showed that MPE prevented the ageing process through downregulation of K-Ras and its downstream targets.

Senescence, an irreversible cell cycle arrest phenomenon, is one of the hallmarks of ageing 1. Cell cycle progression is tightly controlled by cyclin-dependent protein kinases (CDKs) and their negative regulators 21. We demonstrated that MPE increased the association between CDK4/cyclin Ds and CDK2/cyclin E or cyclin A. Moreover, MPE also repressed K-Ras-enhanced CDK inhibitor expression. Quercetin significantly attenuated the expression of p53 in the pancreatic tissues of D-galactose-induced ageing rat 22. Administration of chlorogenic acid dose-dependently suppressed the vascular senescence of C57/BL6 female mice through the downregulation of p53 and p21 expressions 23. Our results indicated that MPE promoted cell cycle progression by controlling the association of CDKs/cyclins and the expression of CDK inhibitors.

AMPK is a key sensor for the determination of cellular energy homeostasis 24. Through regulation of metabolism, AMPK is involved in a wide range of cellular functions, such as apoptosis, autophagy, and longevity 24. Activation of AMPK homologs enhances the transcription activities of Forkhead box protein O (FOXO) and peroxisome proliferator-activated receptor gamma coactivator 1-alpha (PGC-1α) and extended the life span of C. elegans 25 Activation of AMPK by quercetin ameliorated H2O2-induced cell senescence in vascular smooth muscle cells, as evidenced by decreased β-galactosidase activity and repression of the expressions of CDK inhibitors, such as p53, p21, and p16 26. Kou et al. demonstrated that ampelopsin elevated the activity of AMPK/PGC-1α signaling to prevent skeletal muscle atrophy in D-galactose-induced ageing rat 27. In the present study, our results showed that MPE obviously stimulated the activity of AMPK and then diminished the K-Ras-induced senescence of A7r5 cells, whereas the phenomenon was blocked by AMPK inhibitor. Our findings indicated that AMPK played a critical role in MPE-induced anti-ageing processes.

Endothelial dysfunction, which means reduction of the synthesis of endothelial NO, is accompanied by ageing and leads to the development of cardiovascular diseases 28. In the elderly, the inhibition of arginase leads to the elevation of NO-ameliorated endothelial function in an age-dependent manner 29. Ageing-induced NO level was recovered by treatment with (-)-epicatechin (1 μM) for 48 h in primary bovine coronary artery endothelial cells 30. Administration of (-)-epicatechin (1 mg kg-1 day-1) for 15 days also increased the NO in rats compared with the group treated with water alone 30. Herein, we demonstrated that MPE significantly enhanced the activity of iNOS and increased NO level in A7r5 cells stably expressing K-Ras.

## Conclusion

In summary, MPE triggered the activity of iNOS synthase and elevated NO level. NO promoted AMPK activation and attenuated HMG-CoA reductase activity by phosphorylation. MPE blocked Ras-induced senescence of vascular smooth muscle A7r5 cells through the downregulaton of CDK inhibitors and ERK activation. In conclusion, our findings suggested that MPE could be a potential anti-atherosclerosis agent that acts by inhibiting Ras-induced senescence and ageing.
